# Supplementary material for: Estimating individual risks of COVID-19-associated hospitalization and death using publicly available data
Source: PLoS One. 2020 Dec 7;15(12):e0243026. doi: 10.1371/journal.pone.0243026 (PMC7721133; doi:10.1371/journal.pone.0243026)
Supplement: S1 Table — (DOCX) [file pone.0243026.s001.docx]

**S1 Table. Daily contact rates by age and setting.** Contact rates are summed from country and age-specific contact matrixes for the United States supplied in: Prem K, Cook AR, Jit M (2017) Projecting social contact matrices in 152 countries using contact surveys and demographic data. PLoS Comput Biol 13(9): e1005697. <https://doi.org/10.1371/journal.pcbi.1005697>

| Age | Home | Work | School | Other | Non-home | Total |
| --- | --- | --- | --- | --- | --- | --- |
| 20 - 29 Years | 2.77 | 5.39 | 0.94 | 5.55 | 11.88 | 14.66 |
| 30 - 39 Years | 3.20 | 6.09 | 0.80 | 4.22 | 11.10 | 14.30 |
| 40 - 49 Years | 3.18 | 5.92 | 1.11 | 3.47 | 10.49 | 13.67 |
| 50 - 59 Years | 3.33 | 4.61 | 1.70 | 4.22 | 10.53 | 13.86 |
