# Supplementary material for: Estimating individual risks of COVID-19-associated hospitalization and death using publicly available data
Source: PLoS One. 2020 Dec 7;15(12):e0243026. doi: 10.1371/journal.pone.0243026 (PMC7721133; doi:10.1371/journal.pone.0243026)
Supplement: S2 Table — (DOCX) [file pone.0243026.s002.docx]

**S2 Table. Estimates of the case hospitalization and case fatality ratios.** Computed from US CDC surveillance case report data including case report dates from June 16 to September 15, 2020. Computed from: U.S. Centers for Disease Control and Prevention. COVID-19 Case Surveillance Public Data. Available at: <https://data.cdc.gov/Case-Surveillance/COVID-19-Case-Surveillance-Public-Use-Data/vbim-akqf>

| Age | Cases | Hospitalizations | Deaths | CHR | CFR |
| --- | --- | --- | --- | --- | --- |
| 20 - 29 Years | 627819 | 10385 | 285 | 1.65% | 0.05% |
| 30 - 39 Years | 478492 | 14998 | 702 | 3.13% | 0.15% |
| 40 - 49 Years | 426258 | 20251 | 1580 | 4.75% | 0.37% |
| 50 - 59 Years | 389910 | 29134 | 3776 | 7.47% | 0.97% |
