# Supplementary material for: Estimating individual risks of COVID-19-associated hospitalization and death using publicly available data
Source: PLoS One. 2020 Dec 7;15(12):e0243026. doi: 10.1371/journal.pone.0243026 (PMC7721133; doi:10.1371/journal.pone.0243026)
Supplement: S3 Table — (DOCX) [file pone.0243026.s003.docx]

**S3 Table. Cumulative COVID-19 associated hospital admissions rates per 100,000 people during the period June 16 to September 15, 2020**. Computed from US Centers for Disease Control and Prevention COVID-NET program data on laboratory-confirmed Covid-19- Associated Hospitalizations. Available at: <https://gis.cdc.gov/grasp/COVIDNet/COVID19_3.html>

| Age | Period cumulative hospitalization rate |
| --- | --- |
| 18-29 years | 44.4 |
| 30-39 years | 59.2 |
| 40-49 years | 78.5 |
| 50-64 years | 109.1 |
