# Supplementary material for: Estimating individual risks of COVID-19-associated hospitalization and death using publicly available data
Source: PLoS One. 2020 Dec 7;15(12):e0243026. doi: 10.1371/journal.pone.0243026 (PMC7721133; doi:10.1371/journal.pone.0243026)
Supplement: S4 Table — (DOCX) [file pone.0243026.s004.docx]

**S4 Table. Cumulative COVID-19 associated mortality rates per 100,000 people during the period June 16 to September 15, 2020.** Computed from: U.S. Centers for Disease Control and Prevention National Center for Health Statistics Provisional COVID-19 Death Counts by Sex, Age, and Week. Available at: https://data.cdc.gov/NCHS/Provisional-COVID-19-Death-Counts-by-Sex-Age-and-W/vsak-wrfu

| Age | Period deaths | Period cumulative mortality rate |
| --- | --- | --- |
| 15-24 years | 205 | 0.48 |
| 25-34 years | 668 | 1.46 |
| 35-44 years | 1813 | 4.39 |
| 45-54 years | 4499 | 10.81 |
| 55-64 years | 10428 | 24.67 |
