# Supplementary material for: Estimating individual risks of COVID-19-associated hospitalization and death using publicly available data
Source: PLoS One. 2020 Dec 7;15(12):e0243026. doi: 10.1371/journal.pone.0243026 (PMC7721133; doi:10.1371/journal.pone.0243026)
Supplement: S1 Fig — (DOCX) [file pone.0243026.s005.docx]

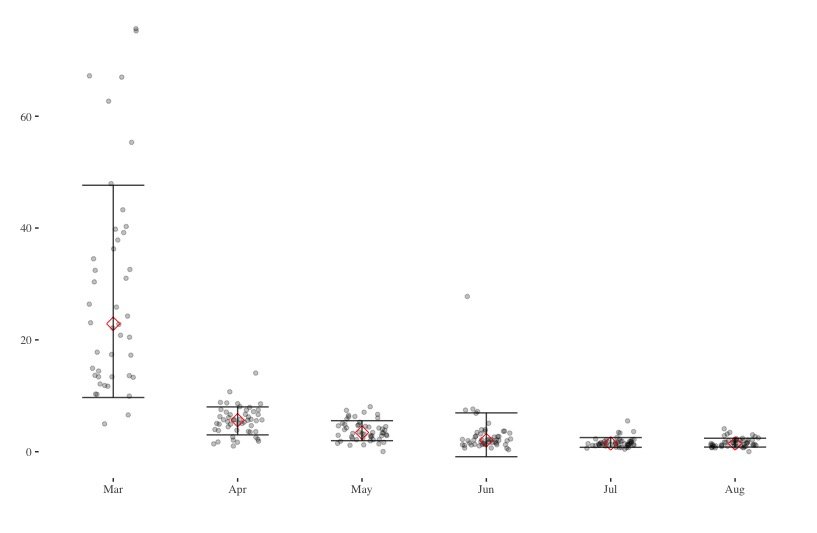


**S1 Fig. Estimates of the state-specific case fatality ratio by month.** Computed from: U.S. Centers for Disease Control and Prevention data. United States COVID-19 Cases and Deaths by State over Time. Available at: https://data.cdc.gov/Case-Surveillance/United-States-COVID-19-Cases-and-Deaths-by-State-o/9mfq-cb36.
